# The data archiving in the small herbaria digitisation workflow

**DOI:** 10.3897/BDJ.14.e188878

**Published:** 2026-03-27

**Authors:** Andriy Novikov

**Affiliations:** 1 State Museum of Natural History of the NAS of Ukraine, Lviv, Ukraine State Museum of Natural History of the NAS of Ukraine Lviv Ukraine

**Keywords:** herbarium digitisation, data storage, data archiving, archiving strategy, long-term storage media, archive managment, small herbaria

## Abstract

The paper provides insight into the archiving of data retained during the digitisation of herbarium materials. It shares practical experience and guidance on best practices for long-term data storage, with particular discussion on storage media and backup strategies. It is aimed at small herbaria that have limited or no dedicated archiving infrastructure and a low budget.

## Introduction

Davis (2023) estimated that there are approximately 400 million herbarium specimens worldwide. This number correlates with the recent report by Thiers (2026), who indicated that over 406 million specimens are deposited in nearly 4000 herbaria. Herbaria serve as an essential and indispensable source of biodiversity data, which are actively mobilised and distributed in a digital format. Digitisation of herbaria provides numerous benefits, including mitigating pressure on physical specimens, making digitised materials accessible remotely and accelerating their involvement in research (Heberling et al. 2019, Paton et al. 2025). Digitised herbarium data are widely used in taxonomic, floristic, ecological, biogeographical and other research. Simultaneously, virtually represented materials also support education and public engagement in science (Heberling 2022, Linnér et al. 2025, Ahlstrand et al. 2025).

There are numerous publications describing the herbarium digitisation workflow and providing insight into crucial aspects of its organisation (e.g. Nelson et al. (2015), Thiers et al. (2016), De Smedt et al. (2024)), including those developed specially for small herbaria usually having limited budget (e.g. Harris and Marsico (2017), Takano et al. (2019)). Nieva de la Hidalga et al. (2020) paid special attention to applied file formats and provided an exhaustive review of the organisation of quality control during herbarium digitisation. It has also been repeatedly emphasised that data collected during digitisation must be findable, accessible, interoperable and reusable; in other words, conform to the FAIR principles (Wilkinson et al. 2016, Lannom et al. 2020). Besides this, stored data must be tidy and, preferably, kept in open and raw formats (Hart et al. 2016).

Data retention is a crucial task that ensures that digitised materials persist in the long term and can be accessed when needed. Nevertheless, the organisation of long-term storage for such data and the management of archived data are discussed only briefly. In large herbaria and institutions with proper financial support, the archiving is often delegated to outsource companies or special departments. However, in small herbaria and limited-budget settings, such delegation can be impossible, leaving herbarium curators to address self-archiving. Most curators organise the archiving, based on their experience and available facilities. In the Herbarium of the State Museum of Natural History of the NAS of Ukraine (LWS), we faced the problem of the archiving several years ago. Many mistakes were made and many lessons were learned since that. Therefore, here I would like to share my considerations on this topic, based on the gained experience.

## General considerations on archival principles and policy

There are no clearly defined requirements regarding how long digitised materials must be preserved. Ideally, digitised herbarium materials, which represent a special kind of data valuable to science and are often in the public domain, should be kept indefinitely. However, from a practical point of view, this means that data should be kept undamaged and stored for at least a few to several decades, ensuring their future migration to other media and/or formats (Lunt 2011, Novikov and Nachychko 2025). Each institution must develop a data leadership infrastructure (Mozzherin and Paul 2023) and data management strategy that describes the expected archival terms, data retention and migration policy. At the same time, institutions must strike a balance between ensuring long-term data storage and the costs and risks associated with storing large volumes of data (e.g. unlawful access or copyright issues). Hence, effective long-term data archiving requires a combination of physical protection, redundancy, integrity verification, documentation and planned technological migration.

There are two types of data retention, depending on the manipulation activity. So-called cold storage means that data are archived and accessed only during planned sessions, usually after a relatively long period (e.g. once per year). Another type is called hot storage, which means that stored data are actively and occasionally accessed and are relatively easily and quickly available all the time. These two retention types are fundamentally different and the choice of archival media and management plan depends strongly on the selected retention type or its combination. For example, if only cold storage of digitised materials is planned, the optimal solution is to use magnetic media such as LTO tapes. Whether there is a need for regular access to archived files or for on-flow completion of the archive, the optimal solution is to use cloud storage or RAID arrays. The choice of the retention type also depends on the available facilities, finances and trained staff. In the absence of such, the better option will be periodic archival, with portions split by digitisation batches. In particular, for biodiversity data in general, Mozzherin and Paul (2023) recommend splitting backups into smaller chunks (e.g. having the size of the largest available long-term storage unit) and separating storage into a read-only (cold) and read/write (hot) sections, implementing the Copy-on-Write resource management technique. This enables the application of cheaper hardware and develops cost-efficient data retention. For the herbarium collections, De Smedt et al. (2024) and Dillen et al. (2024) recommend to combine the outsourced cold storage with internal server-based hot storage.

Retention types also tightly correlate with the backup strategies, which, in turn, depend on the chosen security and fault tolerance levels. In this context, three main archival strategies or so-called 'rules' can be delimited. The *3-2-1 backup strategy* states that at least three copies of data should be kept on at least two types of media, with at least one copy stored off-site (e.g. in the cloud or another institution). The 3-2-1 strategy is the simplest and can be applied in most cases for retaining digitised herbarium data. It assures a strong fault tolerance, but a moderate security level (Ruggiero and Heckathorn 2012, Perkel 2019, Malecki 2021). The other two strategies are advanced, focused on security risks and are mostly redundant for herbarium digitisation. The *3-2-1-1-0 backup strategy* supplements the above requirements with the need to store at least one copy of data on so-called ‘air-gapped’ media, i.e. media that is not directly accessible via the Internet and, accordingly, cannot be damaged by malicious attacks (Stonefly 2026). The ‘air gap’ principle can be implemented both physically (for example, if hard drives are disconnected or stored separately from the main array) and logically (for example, if hard drives are connected to the main array, but access to them is disabled). The 0 in this strategy means it allows no errors, as even a single error can trigger an avalanche of additional errors and, as a result, lead to a fatal data failure. The *4-3-2 backup strategy* focuses more on data protection in the event of a natural disaster. It requires maintaining at least four backup copies of the data on at least three types of media, with one of those copies stored in the cloud. At least two copies should be stored off-site or off-network (for example, on a cloud storage and another facility or on two independent local area networks that are not connected to the Internet). There can be different modifications to the backup strategies depending on institutional needs. For example, a *4-3-2-1 backup strategy* additionally requires one copy to be air-gap stored (BackupChain 2026).

Based on the mentioned above types and strategies, the next archiving key principles can be ascertained: (a) development and following the institutional data management policy; (b) application of durable media and technologies; (c) producing the multiple copies; (d) combination of different media and storage technologies; (e) regular quality control of retained data; and (f) regular data migration and integration with recent technologies.

## File formats

In the case of herbarium digitisation, stored data includes digital images of herbarium specimens (and sometimes their fragments and/or labels), data about these specimens (in most cases, the data gathered from the herbarium labels) and metadata (mostly describing the origin of the data, legal aspects of distribution, the digitisation process etc.). Besides this, the principal data can be supported by additional data (e.g. cryptographic or non-cryptographic hashes), simplifying further quality checks (Hart et al. 2016). Park and Oh (2012) comprehensively examined various open file formats commonly used for archival purposes. They found that key features of such file formats include their functionality, openness, interoperability, independence and the ability to provide extended metadata. These conclusions are consistent with the FAIR principles (Wilkinson et al. 2016, Lannom et al. 2020), as well as Library of Congress (2026) recommended formats and FADGI requirements (Rieger et al. 2023).

**Figure 1.**
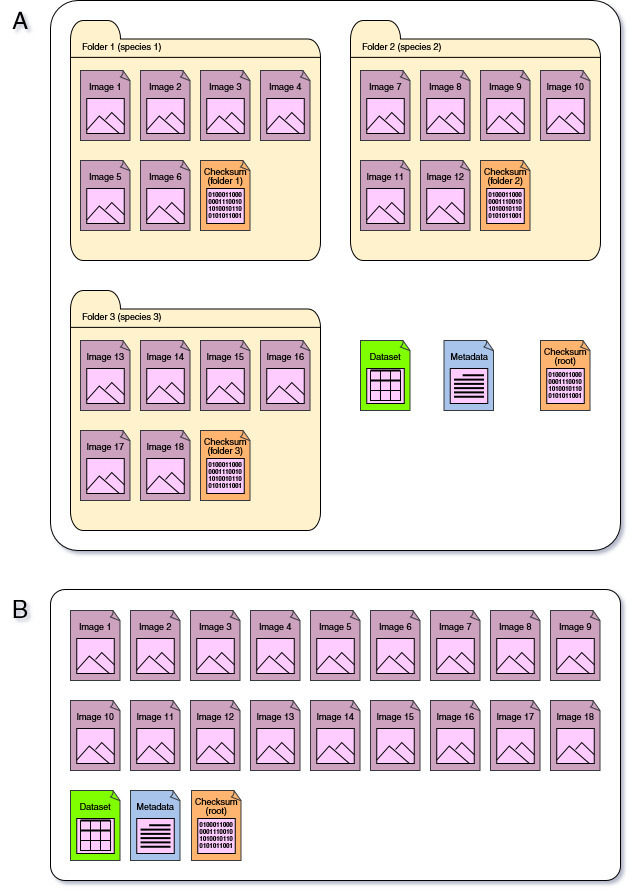
Variants of the file organisation applied in the LWS herbarium archiving. **A** JPG files named by specimens' IDs and sorted by folders named by species/infraspecies; **B** original RW2 files stored in bulk.

Therefore, each archiving set should contain the following files: (a) images of the digitised specimens; (b) dataset with data on respective specimens; (c) metadata and (d) checksum. In turn, these files can be stored in bulk in a root folder or organised by folders (e.g. by species/infraspecies name). The organisation of files into folders is controversial. In small herbaria, it can be beneficial for human operation (e.g. navigation and sorting), but in general, it is problematic for machine operation. Moreover, creating the folders and sorting the images requires extra time and effort. At the same time, if digitisation is organised in batches, it may result in folders with the same name being stored on different volumes of archival media. Therefore, organisation of files in folders must be carefully considered. The general logic of folder and file structure applied in the LWS herbarium, is represented in Fig. 1.

### Digital images

There are three main types of digital images produced during the herbarium digitisation (Nieva de la Hidalga et al. 2020): original master files, derivative lossless images and derivative lossy images. However, as our practice showed (Novikov and Nachychko 2025), it is faster to produce master files in RAW format and lossy images in JPEG format directly from the camera, omitting the extra step of producing lossless images. In the LWS Herbarium, these two file types are archived simultaneously. Derivative images in lossy formats are normally not used for archiving, as they can always be produced from the original master files or from lossless formats. However, if the facilities allow, archiving the derivative files can be useful because it increases the number of stored copies.

*Master files* are typically represented in RAW or TIFF formats and originate directly from a camera or scanner. They serve as principal files for long-term storage. The derivative images are stored in converted target formats that depend on their intended use. *Images in lossless formats* are usually used for internal applications and where high resolution is required. For herbarium materials, images are stored in TIFF or JPEG2000 formats, which preserve original image quality, while still slightly compressing the files. Images in lossless formats are also often available for download from virtual herbaria, but preliminary display is usually done with heavily compressed lossy images. Such splitting of the functions of display and download allows for reducing server load and speeding up online operations with virtual herbaria. *Images in lossy formats* are usually stored as JPEGs. These images are used for most regular operations because they are significantly smaller than master files and lossless derivative files (Table 1). However, converting original files received directly from the camera or scanner does not always reduce file size. Sometimes re-saving the file, even in the same format, can increase its size (Table 1). It is also worth noting that, technically, files in both TIFF and JPEG2000 formats can be stored in lossy format, with considerable compression and consequent quality losses. However, it happens extremely rarely since there is no evident reason or benefit to store lossy files in such formats.

Before archiving, image files must be renamed using the unique herbarium specimen IDs. For this purpose, it is best to use automatic renamers (e.g. the online renamer deposited at herbUA (Novikov 2026) or BCRWatcher (Lafferty 2026)), which read barcodes from images and rename the files accordingly. However, such renamers usually do not work with RAW formats. Therefore, master files should either be kept as is or manually renamed. There is also an option to create an additional table matching the original file names and the renamed JPEG file names and apply it to RAWs, since RAWs usually keep the same naming.

### Specimens' data

Data structure and presentation can differ significantly depending on the source (e.g. generated from the Specify database) or the applied standard (e.g. Darwin Core). Regardless of the applied model, the data about the herbarium specimens are usually represented as a dataset that can be saved in various formats. For dataset archiving, it is recommended to use well-known, publicly validated formats (e.g. CSV or TSV) with UTF-8 character encoding (Hart et al. 2016, Library of Congress 2026). Open-specification data formats can be processed in many programming languages, since efficient, well-tested parsing libraries are usually widely available. Other well-recognised formats (e.g. XLSX) and character encodings (e.g. Windows-1252) can also be applied, but in such cases, they must be clearly stated in the associated metadata. In the case of the LWS Herbarium, the core dataset is represented in TSV format following the Darwin Core (TDWG 2026) standard.

### Metadata

Metadata for digital images can be embedded in their files or stored in a separate file. Other associated metadata can also be integrated into the main dataset or represented as a separate file. For example, data downloaded from GBIF as Darwin Core Archive combining the main dataset in TXT format, optional extension data file (e.g. file containing links to digital images and respective atributes) in TXT format, metafile describing relationships between the files (present only in case of extension files) in XML format and metadata file describing this dataset in XML format (GBIF 2026a). The same logic should be applied to archiving digitised herbarium materials, regardless of whether they are planned for publication in GBIF. In the case of the LWS Herbarium, the metadata are represented in XML format following the EML standard.

### Checksums

For the retention of digitised herbaria, there is normally no need to apply cryptographic hashes (e.g. MD5 or SHA256), as the risk of malicious or unauthorised access to the data is low. In such a case, it is sufficient to use a non-cryptographic checksum, such as CRC32, which generates a unique 32-bit integer for each file or file set. Such integers are useful for quickly detecting accidental file corruption and are recommended for archival purposes. For example, the open-source software RapidCRC (2005) can generate both CRC32 and MD5 checksums, which can be stored along with the main archiving files (i.e. digital images, dataset and metadata). In the case of the LWS Herbarium, CRC32 checksums are applied to each folder and stored in SFV format.

## Archiving media

For long-term data storage, FADGI (Rieger et al. 2023) recommends using RAID hard drive arrays with cyclic redundancy check (CRC) error correction. For herbarium digitisation, Haston et al. (2012) recommend using multiple types of physical media simultaneously (e.g. magnetic tapes and external hard drives). If archiving is performed on the same type of media, using media from different manufacturers is also advisable to avoid possible manufacturing defects and potentially low production quality (Rieger et al. 2023).

Although FADGI does not recommend using *optical media* for long-term data storage, they can still be considered a good choice due to their relative longevity in a controlled storage environment and cost value (Brown 2008, ISO 2018). Optical media are also considered reliable for archiving purposes due to their simplicity (no mechanical or electronic components) and relatively high resistance to electromagnetic interference (Gu et al. 2014, Wan et al. 2015). Of course, optical media have drawbacks, as they can degrade relatively easily when exposed to direct sunlight or heat (Slattery et al. 2004). Failure of optical media also strongly depends on the track pitch; the smaller the pitch, the shorter the retention period. Special optical media like M-discs or Blu-ray discs, resistant to physical influence, can be considered an effective WORM ('write once, read many') storage media for data retention in case of a relatively small amount of required space (up to several terabytes) and the need for hot storage. These optical discs assure long-term storage and reproduction of the data for decades, even hundreds of years (Svrcek 2009, Petrov et al. 2011, Iraci 2019, Mozzherin and Paul 2023). However, if there is a need to store much more data and no need to access it frequently or quickly, LTO cassettes seem to be the only solution for cold storage.

*Magnetic media* offer extremely high storage capacities at a low price. Modern magnetic cassettes can store up to 18 TB (LTO-9) or even 30 TB (LTO-10) of data and ensure data retention for 30-50 years. However, magnetic media also have weaknesses: they are sensitive to electromagnetic radiation and are usually enclosed in special electromechanical 'envelopes', which can also fail. Such magnetic media as LTO tapes offer superb price-to-volume value and longevity, but can be used only for cold storage, as they have limited rewriting potential (Wan et al. 2015, Lantz et al. 2025). The data on the magnetic tapes can be accessed only sequentially (data are read one at a time), which slows the process. For comparison, data on HDDs, another type of magnetic storage media, can be accessed at any time. Direct access to data saves time and prevents other portions of the HDD magnetic surface from being used and, hence, from losing their working potential. Nevertheless, HDDs have additional electrical and mechanical components, complicating their construction and, as a result, reducing overall reliability (Henriksen et al. 2013). As mechanical components are present, HDD discs can be easily damaged by shock from drops. HDDs have a limited lifetime (typically ca. 20 years) and, besides regular data migration, require extra control, which is cost- and energy-consuming (Gu et al. 2014, Bhat 2018). A combination of optical or HDD media to store the derivative files in lossy format and LTO cassettes to store master files also seems viable, as the lossy files are relatively small and are usually more frequently accessed, while master files are larger and accessed only as needed. A similar archiving strategy is also applied at Meise Botanic Garden (De Smedt et al. 2024), which keeps JPEG and JPEG2000 files on internal servers while cold-storing TIFFs on an LTO-based outsourced storage system.

*Electronic storage media* (e.g. flash drives and SSDs) depend heavily on electronic components, have limited rewrite cycles and can lose data over time due to wear. In general, electronic storage media are not suitable for long-term archiving and also have relatively low fault tolerance due to active degradation during use (Cai et al. 2015). Although modern electronic storage media use mainly non-volatile architecture, they still gradually lose information due to the discharge of memory cells without additional power and require specialised error-correction and data versioning algorithms (Zhu et al. 2025). In particular, the currently popular SSDs, based on NAND flash memory, gradually lose data after 2 years when not connected to a power source. Electronic storage media also have indirect heat intolerance. When the temperature increases or decreases, the storage period of information significantly reduces because flash memory loses its charge faster (Cox 2015, Patrizio 2015). To overcome this drawback, electronic media with built-in batteries were constructed. However, even such media, as a rule, cannot store information for more than 5 years without an additional power source. In addition to the aforementioned disadvantages, electronic storage media are complicated in their architecture and quite expensive. The cost of storing 1 gigabyte of data on a flash drive can be much higher than that of storing the same amount on a traditional hard drive.

*Cloud storage services* (e.g. ShareArchiver (2026), Azure Blob Storage (Microsoft (2026)), Blackblaze (2026), CyVerse (2026)) are also often used for data archiving. At its core, cloud storage also uses physical storage media (hard drives), but thanks to its robust backup system, it is one of the most reliable options for long-term data storage. However, it should be noted that such resources are commercial and the storage period directly depends on the paid period. Cloud storage services are an attractive solution if there are no archiving facilities and/or experience in the institution. Delegation of archiving to an outsourced provider can be beneficial, as it can save time and costs on developing one's own infrastructure and training personnel. However, being commercial companies, cloud storage services are not secured from the risk of economic bankruptcy. Additionally, copyright and other legal aspects should be carefully considered when cooperating with outsourced companies. Moreover, when relying on outsourced companies, it is important to ensure they are actually delivering the services you expect and to maintain ongoing control.

In the LWS Herbarium, data archiving follows the 3-2-1 rule (Novikov and Nachychko 2025). In particular, data are stored on two principal types of media: (a) hard drives of the internal server of the State Museum of Natural History of the NAS of Ukraine; (b) Blu-ray MABL discs. Additionally, SD memory cards serve as test storage media to analyse how long the data will persist. Normally, SD memory cards are used as temporary storage media for quick file sharing, serving as a plug-and-play solution since not all modern computers can read optical discs. In addition, the LWS Herbarium data are archived on Zenodo (CERN 2026) and distributed through GBIF (2026b), Open Herbarium (2026), herbUA (Novikov 2026) and other online services. Due to the increasing number of files, starting in 2027, it is planned to introduce cold data archiving in the LWS Herbarium using LTO cassettes.

## Archive management

Archiving must be deployed to qualified personnel and implemented in a controlled environment because, in addition to technical issues, human-driven failures are a significant concern (Li et al. 2012). Mozzherin and Paul (2023) noted that data leadership must be developed within research institutions working with data. Ideally, such leaders should hold permanent positions and have clearly defined roles, including data retention. In particular, Stack and Stadolnik (2018) designate four principal roles within the unified data leadership: data manager, analytics oficer, data scientist and chief data officer. In this context, Dillen et al. (2024) identified four roles within the Meise Botanic Garden Herbarium data management infrastructure, based on key responsibilities: image manager, database manager, portal manager and scientific manager. Within this infrastructure, image and database managers are responsible for data retention. Dillen et al. (2024) also highlighted the need for herbarium-hosting institutions to develop data management plans, not only for data retention, but also to ensure data usability, integrity and security. Hence, the best practice is to delegate archiving to the specialised data department and/or manager, who are set up for this purpose within the data leadership infrastructure. However, in small herbaria, it can be impossible due to the lack of such infrastructure and the costs for its development. Often in small herbaria, all data management is concentrated in the hands of a single curator or custodian.

With limited resources, it is important to create as controlled an environment as possible for data storage. In particular, it is necessary to designate a place (e.g. a cabinet) where the archival media will be stored, with minimal exposure to negative factors. The storage place must have a clear and visible indication so it can be prioritised for evacuation in case of an emergency. The access to the storage place preferably must be delegated to one responsible person. However, organisation of the archive, as well numbering and labelling of media must be clear and understandable for a wide audience. All manipulations with the archive must be trackable.

Data must be prepared for long-term storage and pass basic pre-archival preparation. The set of audits allowing receipt of the data and images of required quality during the herbarium digitisation is comprehensive and thoroughly discussed by Nieva de la Hidalga et al. (2020). Besides this, the brief check of digitised materials just before archiving (writing on the archival media) can be helpful. Such check can be organised as a questionnaire, as was done in the LWS Herbarium (Table 2). Further quality control shall include periodic (with one-year interval) examination to ensure the lack of failures in recorded data (ISO 2018). For these purposes, special software (e.g. RapidCRC) and checksums can be applied. In addition to checking the files' integrity, visually inspecting archiving media for signs of damage and/or degradation is also applicable.

Despite the data migration being a non-obvious task at first glance, it is also crucial for their successful retention. Shifts in storage technologies and the gradual degradation of storage media must be taken into account, which is why data migration is typically carried out every three to five years (Hodge 2000, SWGDE 2026). In case of a limited budget, such a migration can be costly, so it can be extended to 10 years, with an audit midway through. The extended audit, besides the analysis of stored media and data, must examine the current state of: (a) the applied archiving technology and its perspectives; (b) the reading/writing hardware, including its working conditions, repairability and presence of hardware and its components on the market/aftermarket; (c) the applied file formats and its perspective of use in the near future. In cases where keeping old technology is considered, an additional financial audit comparing the costs of old technology vs. migrating to a new one can also be useful.

## Some tips and tricks we learned

De Smedt et al. (2024) shared the ten lessons learned during the digitisation of the Herbarium of the Meise Botanic Garden (BR). These lessons must be read by everyone planning the digitisation of the herbarium collection, regardless of its size or budget, to help avoid mistakes. Here, I would like to extend the presented suggestions with the lessons we learned during the archiving of our digitised materials, with the hope that they will be helpful to other herbarium curators:

1. The archiving strategy is a crucial step to success and must be developed before the start of the work. A properly developed strategy will save time, effort and money. An improperly developed strategy will allow for documenting the mistakes and adapting in the future. On the other hand, this is time-consuming and may be more expensive, as you may need to buy additional equipment or materials.
2. The archiving strategy must align with a broader data management plan, ensuring data interoperability, distribution, preservation, integrity and security.
3. Something is better than nothing. In emergency situations (e.g. during hostilities), there is no time to develop strategies or learn data archiving. In such a case, any kind of digitisation and data archiving will be appreciated. It may take longer to proceed with such raw data, but it may be the only data that survive harsh times.
4. Choose those archiving media and technologies as simply as possible. It may include facilities more common in your region and/or more widely used in the specific institution. It will help to synchronise efforts and obtain help from colleagues.
5. Do not be afraid of the aftermarket. In cases of a minimal budget, it may be better to buy archiving hardware and media on the aftermarket. Acquiring used, refurbished or stock equipment is a cost-effective option. However, the aftermarket is inherently risky, so such a purchase must be made by a qualified person.
6. Keep the documentation as open as possible and share your experience with colleagues. So, if needed, another person could take over the digitisation and archiving.
7. Data retention cannot be delegated to inexperienced and temporary staff. The best solution is to identify one person or group to take responsibility for the data. In small herbaria, unfortunately, it is usually the same person who cares for the herbarium.
8. Even when delegating the archiving to an outsourced company, learn the basics. This will help you choose the company best suited to your interests and, if needed, you can take over the archiving process.
9. Take a look at the future. Even if the archiving strategy you've applied seems good for you, it may become insufficient in the near future. The amount of data can become so large that it becomes problematic to store it on small media.
10. Keep the raw images as raw as possible. Do not apply any transformations to save space besides trimming the empty space. Trimming the empty space around the herbarium sheet in the images can significantly reduce the file size.
11. Use the same file formats as long as it is possible. Even changing the file extension from .jpg to .jpeg can cause issues with automatic processing (e.g. hyperlinks, if applied, will no longer work). However, do not be afraid to migrate to the new file format if there are justified reasons (e.g. the old file format is no longer supported or weakly supported by newer machines).

## Conclusions

Herbaria serve as natural history archives and aim to save the herbarium material for as long as possible. The digitisation of deposited materials extends this period virtually. However, all digitisation efforts may be in vain if long-term data storage is not taken care of. Data retention in small herbaria can be tricky and depends heavily on available expertise and facilities. However, it cannot be ignored or under-evaluated, as correct retention depends on how long data will be preserved and how easily it can be retrieved in case of need. Developing a robust archiving strategy and selecting appropriate archiving media can significantly increase the likelihood that data will survive. The optimal solution for small herbaria is to use the 3-2-1 backup strategy, which aims to produce three copies of data stored on two types of media, with one copy deposited outside the institution. The combination of magnetic (HDD and/or LTO) and optical media (Blu-ray discs) can be a good choice, ensuring data preservation for at least 10 years. Nevertheless, regular data quality control and data migration must be included in the archive management plan.

## Figures and Tables

**Table 1.** Comparison of file sizes of images saved in different formats. The original digital images were captured with the Panasonic Lumix DC-G9 camera, which features built-in pixel-shift technology. Adobe Camera Raw 12.2.1 was used to create the derivative files.

**Origin**	**Compression**	**File format**	**Resolution, MP**	**File size, MB**
Original (from camera)	Lossless	RAW (.rw2)	80	125
Original (from camera)	Lossy	JPEG (.jpg)	40	18
Derivative	Lossless	DNG (.dng)	80	153
Derivative	Lossless	TIFF (.tiff)	80	461
Derivative	Lossless	JPEG 2000 (.jpf)	80	64.1
Derivative	Lossy	TIFF (.tiff), JPEG commpression	80	41.6
Derivative	Lossy	JPEG 2000 (.jpf), DWT wavelet	80	8.7
Derivative	Lossy	PNG (.png)	80	360
Derivative	Lossy	JPEG (.jpeg), standard mode	80	37.5
Derivative	Lossy	JPEG (.jpeg), progressive mode	80	33.9
Derivative	Lossy	GIF (.gif), 256 web colors, normal mode	80	20

**Table 2.** Pre-archiving audit of the folders and files at the LWS Herbarium.

Audit trail	Question	Checkbox
Folder	The folder structure corresponds to the designated folder	
	There are no empty folders	
	There are no excessive folders	
	Each folder is named appropriately (by the species/infraspecies name)	
	There are no hidden folders and/or files	
	Each folder contains the set of images	
	Each folder (including the root folder) contains the checksum file	
	Root folder contains dataset file	
	Root folder contains the metadata file	
Image files	All files are displayed correctly in preview mode	
	All files have the same format (RW2/JPG depending on the archiving preferences)	
	All images have the same (vertical) orientation	
Dataset file	Dataset file saved in TSV format	
	Dataset file is operable (try to open it)	
Metadata file	Metadata file saved in XML format	
	Metadata file is operable (try to open it)	
Checksums	Checksum files show no errors (open each checksum file in RapidCRC and run test)	
